# First application of data assimilation-based control to fusion plasma

**DOI:** 10.1038/s41598-023-49432-3

**Published:** 2024-01-17

**Authors:** Yuya Morishita, Sadayoshi Murakami, Naoki Kenmochi, Hisamichi Funaba, Ichihiro Yamada, Yoshinori Mizuno, Kazuki Nagahara, Hideo Nuga, Ryosuke Seki, Masayuki Yokoyama, Genta Ueno, Masaki Osakabe

**Affiliations:** 1 Department of Nuclear Engineering, Kyoto University, Kyoto, Japan; 2 National Institute for Fusion Science, National Institutes of Natural Sciences, Toki, Japan; 3 The Institute of Statistical Mathematics, Research Organization of Information and Systems, Tachikawa, Japan; 4 The Joint Support-Center for Data Science Research, Research Organization of Information and Systems, Tachikawa, Japan; 5 Department of Fusion Science, The Graduate University for Advanced Studies, SOKENDAI, Toki, Japan; 6 Statistical Science Program, The Graduate University for Advanced Studies, SOKENDAI, Tachikawa, Japan

**Keywords:** Nuclear energy, Computational science

## Abstract

Magnetic fusion plasmas, which are complex systems comprising numerous interacting elements, have large uncertainties. Therefore, future fusion reactors require prediction-based advanced control systems with an adaptive system model and control estimation robust to uncertainties in the model and observations. To address this challenge, we introduced a control approach based on data assimilation (DA), which describes the system model adaptation and control estimation based on the state probability distribution. The first implementation of a DA-based control system was achieved at the Large Helical Device to control the high temperature plasma. The experimental results indicate that the control system enhanced the predictive capability using real-time observations and adjusted the electron cyclotron heating power for a target temperature. The DA-based control system provides a flexible platform for advanced control in future fusion reactors.

## Introduction

Magnetic confinement fusion is a promising next-generation power source. To generate fusion-based power, the fusion plasma should be heated to attain and maintain a good state of confinement. However, fusion plasma is a typical complex system in which various physical quantities interact with each other to determine the overall behavior^1^. Therefore, a large number of variables should be considered for controlling the fusion plasma. An adaptive system (predictive) model is required to account for the latent variables that are difficult to observe or model (e.g., wall conditions^2^) along with uncertain elements related to stability, abrupt termination events, and energy and particle transport. These challenges exist in common with the control of complex systems.

Conventional controllers (e.g., proportional-integral-derivative (PID) controllers) exert simple control over systems with few variables. However, various controllers and observers should be combined to control nonlinear complex systems with large uncertainties and numerous variables. Consequently, the control system becomes extremely complicated and considerable effort is required to construct a well-coordinated control system. For such nonlinear systems, adaptive model predictive control is computationally expensive and difficult to achieve^3^.

Aiming to overcome these challenges, we developed a control system based on data assimilation (DA) techniques^4,5^. In the DA framework, the state of the system is expressed as a probability distribution of the state vector (state distribution), which enables the calculation of the time evolution and conditional probability distribution (information imposition) of the system. Here, the state vector expresses the state of the target system and includes physical quantities and model parameters as components. In general, DA is employed to estimate unobservable variables and models^6–9^ and optimize the model parameters for higher prediction accuracy^10–12^. This study used DA for state estimation (including the model parameters) and control estimation to achieve adaptive model predictive control. This approach facilitates the representation of multiple state variables, observations, and control objects within a single framework, thereby simplifying the control system. In addition, control constraints and domain knowledge can be incorporated into the system model and state variables. The proposed DA-based control system describes the system model adaptation and control estimation from a unified perspective based on state distribution and offers a flexible control platform for the advanced control of fusion reactors.

Recently, methods implementing data-driven approaches for predicting and controlling fusion plasmas, including the use of deep and reinforcement learning, have been investigated^13–15^. The data-driven approach is an inductive approach that constructs predictive and control models based on the given data. It facilitates flexible modeling and highly accurate approximations of target systems within the training data. Contrarily, it requires a large amount of training data and often has low prediction accuracy outside of the data. In addition, the training process is performed again for different scenarios. The physics-based approach, which is a deductive approach based on governing equations, is understandable and tractable by humans. However, it often fails to approximate real systems, particularly complex systems, with high accuracy. The DA-based control approach has the properties of both the data-driven and physics-based approaches. It can improve the prediction performance of the physical model using observation data and capture the internal state and control processes of the system. The proposed approach also enables the construction of a device-independent control system.

The proposed DA-based control system was implemented to control the plasma in the Large Helical Device (LHD), which is one of the largest superconducting plasma confinement devices in the world^16,17^, located in Japan. The effectiveness of this system was verified by performing a simple control experiment. The helical device was desirable for the first demonstration experiment because of its stable magnetic field. In the experiment, the control system estimated the power of the electron cyclotron heating (ECH) required to deliver the target electron temperature. To bridge the gap between the system model and actual behavior, the sequentially observed electron temperature and density profiles were assimilated into the system model. This study is the first to demonstrate real-time DA and adaptive predictive control based on the DA of fusion plasmas. The proposed control scheme is applicable to fusion plasmas and other complex systems (e.g., traffic control, infectious disease control measures, and river level control), and is expected to advance complex system control.

**Figure 1:**
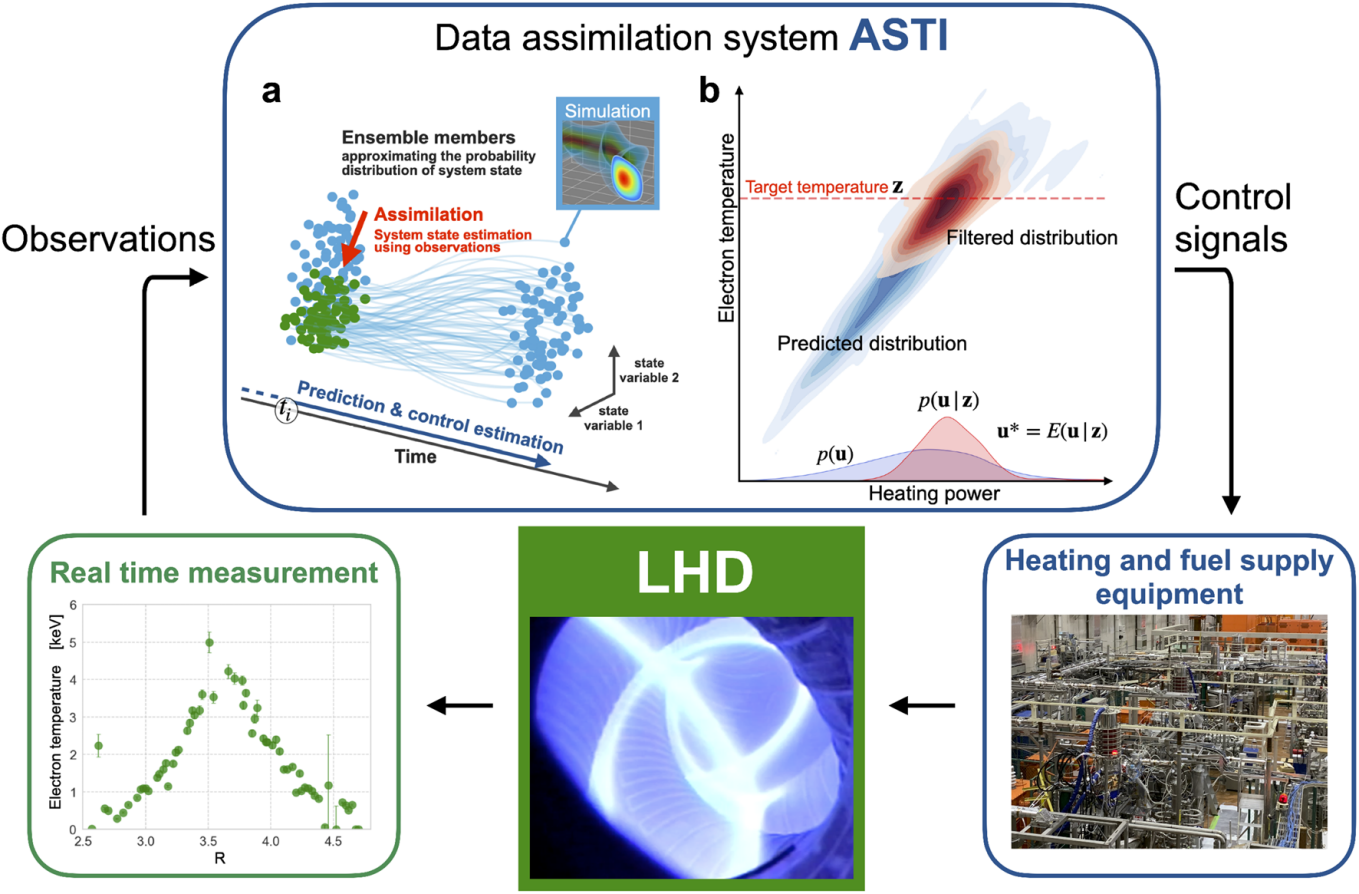
Overview of the DA-based control system for fusion plasmas. (**a**) The state distribution in the DA system (ASTI) is expressed as an ensemble. The parallel time evolution of each ensemble member approximates that of the state distribution. (**b**) The control estimation is performed by assimilating the target state into the predicted state distribution.

## Methods

Figure 1 shows a conceptual diagram of the DA-based control system. This system has a simple structure comprising a DA system, heating and fuel supply equipment, and measurement devices. This section describes the DA-based control system constructed for the LHD plasma control.

### DA framework for adaptive predictive control

The DA system, which is the core component of the control system, computes the probability distribution of the state vector (state distribution), which is defined at time *t* as $\textbf{x}_{t}=(\tilde{\textbf{x}}_{t}^{\textrm{T}}, \textbf{u}_{t}^{\textrm{T}})^{\textrm{T}}$, where vector $\tilde{\textbf{x}}_{t}$ is the part of $\textbf{x}$ that includes the system state and model parameters, and vector $\textbf{u}$ is the control input that determines the time evolution of the system (see Table 1 for an example). The superscript “T” denotes the transpose of a vector and $\Delta t_z$ is the time interval for estimating the control inputs. The DA system optimizes $\tilde{\textbf{x}}$ based on the observed information $\textbf{y}$ (adaptation) and estimates $\textbf{u}$ based on the predicted state distribution and target state $\textbf{z}$ (predictive control).

Consider a control problem in which the control input is adjusted at each time interval $\Delta t_z$ and the system state is observed as the vector $\textbf{y}$ at each time interval $\Delta t_y$. For simplicity, assume that $\Delta t_y = n\Delta t_z$ ($n\in \textbf{N}$) and use the time notation $(i,j)=t_{i,j}=t_0+i\Delta t_y + j\Delta t_z$ and $t_i = t_{i,0}$, where $t_0$ denotes the initial time. The DA framework for adaptive predictive control^18^ is based on the following state-space model:

1 $$\begin{aligned} \textbf{x}_{(i,j+1)}= & {} f_{(i,j+1)}(\textbf{x}_{(i,j)},\ \textbf{v}_{(i,j+1)}), \end{aligned}$$

2 $$\begin{aligned} \textbf{z}_{(i,j)}= & {} H^\textbf{z} \textbf{x}_{(i,j)}+ \textbf{w}^\textbf{z}_{(i,j)},\end{aligned}$$

3 $$\begin{aligned} \textbf{u}^*_{(i,j)}= & {} H^\textbf{u}{} \textbf{x}_{(i,j)}+ \textbf{w}^\textbf{u}_{(i,j)},\end{aligned}$$

4 $$\begin{aligned} \textbf{y}_{i}\ \ \ {}= & {} H^\textbf{y} \textbf{x}_{(i,0)}+ \textbf{w}^\textbf{y}_i. \end{aligned}$$

Equation (1) represents the system model that describes the time evolution of the system $\textbf{x}_{(i,j)}\rightarrow \textbf{x}_{(i,j+1)}$, considering the effect of system noise $\textbf{v}_{(i,j+1)}$. Assume that the value of $\textbf{u}_t$ is constant in the prediction interval $\Delta t_z$, i.e.,

5 $$\begin{aligned} \textbf{u}_{(i,j+1)}= & {} \textbf{u}_{(i,j)} + \textbf{v}^\textbf{u}_{(i,j+1)},\end{aligned}$$

6 $$\begin{aligned} \tilde{\textbf{x}}_{(i,j+1)}= & {} \tilde{f}_{(i,j+1)}(\tilde{\textbf{x}}_{(i,j)},\ \textbf{u}_{(i,j+1)},\ \tilde{\textbf{v}}_{(i,j+1)}). \end{aligned}$$

Here, the system noise for the control input $\textbf{v}^\textbf{u}_{(i,j+1)}$ is added to $\textbf{u}_{(i,j)}$ before calculating the time-evolution. Equations (2)–(4) represent the relationship between the state vector $\textbf{x}_{(i,j)}$ and vectors $\textbf{z}_{(i,j)}$, $\textbf{u}_{(i,j)}^*$, and $\textbf{y}_{i}$ based on the noises $\textbf{w}^\textbf{z}_{(i,j)}$, $\textbf{w}^\textbf{u}_{(i,j)}$, and $\textbf{w}^\textbf{y}_{i}$, respectively. The matrices $H^\textbf{z}$, $H^\textbf{u}$, and $H^\textbf{y}$ are linear operators for projecting the state vector in each corresponding space. The system noise $\textbf{v}_{(i,j+1)}$ is assumed to follow a Gaussian distribution with zero mean and covariance matrix $Q_{(i,j+1)}$, i.e., $\textbf{v}_{(i,j+1)} \sim N(\textbf{0}, Q_{(i,j+1)})$. Similarly, $\textbf{w}^\textbf{z}_{(i,j)}$, $\textbf{w}^\textbf{u}_{(i,j)}$, and $\textbf{w}^\textbf{y}_i$ are assumed to follow the probability distributions $N(\textbf{0},R^\textbf{z}_{(i,j)})$, $N(\textbf{0},R^\textbf{u}_{(i,j)})$, and $N(\textbf{0},R^\textbf{y}_i)$, respectively. The priority of the controlled and observed variables can be adjusted using $\textbf{w}^\textbf{z}_{(i,j)}$ and $\textbf{w}^\textbf{y}_{i}$.

In the DA system, the state distribution is approximated by an ensemble using parallel computing. Each ensemble member represents a simulation with slightly different conditions (e.g., initial conditions, model parameters, and control inputs). In this experiment, considering the time required for computation and communication, the prediction and control estimations were performed up to $\Delta t_y$ ahead of the real time at $n=3$ ($\Delta t_y = 3\Delta t_z$). The following are the computational steps for the adaptive predictive control:

- Prediction
  7 $$\begin{aligned} p\Big (\textbf{x}_{(i,j)}\mid \textbf{y}_{0:i-1},\textbf{u}^*_{(0,1):(i,j)} \Big ) \rightarrow p \Big (\textbf{x}_{(i,j+1)}\mid \textbf{y}_{0:i-1},\textbf{u}^*_{(0,1):(i,j)} \Big ). \end{aligned}$$
- z-filter
  8 $$\begin{aligned} p\Big (\textbf{x}_{(i,j+1)}\mid \textbf{y}_{0:i-1},\textbf{u}^*_{(0,1):(i,j)} \Big ) \rightarrow p \Big (\textbf{u}_{(i,j+1)}\mid \textbf{y}_{0:i-1},\textbf{u}^*_{(0,1):(i,j)},\textbf{z}_{(i,j+1)} \Big ). \end{aligned}$$
- u-filter
  9 $$\begin{aligned} p \Big (\textbf{x}_{(i,j+1)}\mid \textbf{y}_{0:i-1},\textbf{u}^*_{(0,1):(i,j)} \Big ) \rightarrow p \Big (\textbf{x}_{(i,j+1)}\mid \textbf{y}_{0:i-1},\textbf{u}^*_{(0,1):(i,j+1)} \Big ). \end{aligned}$$
- y-filter
  10 $$\begin{aligned} p \Big (\textbf{x}_{(i+1,0)}\mid \textbf{y}_{0:i-1},\textbf{u}^*_{(0,1):(i+1,0)} \Big ) \rightarrow p \Big (\textbf{x}_{(i+1,0)}\mid \textbf{y}_{0:i},\textbf{u}^*_{(0,1):(i+1,0)} \Big ). \end{aligned}$$

here the subscript $t_1:t_2$ denotes all the timings in $[t_1,t_2]$. Given the distribution $p(\textbf{x}_{(i,j)}\mid \textbf{y}_{0:i-1},\textbf{u}^*_{(0,1):(i,j)})$, the prediction step computes $p(\textbf{x}_{(i,j+1)}\mid \textbf{y}_{0:i-1},\textbf{u}^*_{(0,1):(i,j)})$, the distribution $\Delta t_z$ ahead, based on the system model. This step can be performed by computing the time evolution of each ensemble member approximating the state distribution.

The z- and u-filter are the control estimation steps, and the y-filter is the adaptation step. These three steps are based on the Bayesian filter, which calculates the conditional probability distribution $p(\textbf{x}\mid {\xi })$ using the distribution $p(\textbf{x})$ and the model representing the relationship between $\textbf{x}$ and the imposed information ${\xi }$, ${\xi }=h(\textbf{x})+\textbf{w}$. Here, *h* represents the relationship between ${ \xi }$ and $\textbf{x}$ and $\textbf{w}$ represents the associated noise. Generally, $\xi$ denotes the observation data, and the Bayesian filter can assimilate the observed information into the state distribution. Kalman filter (EnKF)^19^ and particle filter (PF)^20^ are typical computational methods for the Bayesian filter that use an ensemble.

The z-filter step estimates the control input by assimilating the target $\textbf{z}_{(i,j+1)}$ into the predicted distribution based on the Bayesian filter using Eq. (2),

11 $$\begin{aligned} p \Big (\textbf{x}_{(i,j+1)}\mid \textbf{y}_{0:i-1},\textbf{u}^*_{(0,1):(i,j)} \Big ) \rightarrow p \Big (\textbf{x}_{(i,j+1)}\mid \textbf{y}_{0:i-1},\textbf{u}^*_{(0,1):(i,j)},\textbf{z}_{(i,j+1)} \Big ), \end{aligned}$$

and performing marginalization

12 $$\begin{aligned}{} & {} p \Big (\textbf{u}_{(i,j+1)}\mid \textbf{y}_{0:i-1},\textbf{u}^*_{(0,1):(i,j)},\textbf{z}_{(i,j+1)} \Big ) \nonumber \\{} & {} \quad = \int p \Big ({\textbf{x}}_{(i,j+1)}\mid \textbf{y}_{0:i-1},\textbf{u}^*_{(0,1):(i,j)},\textbf{z}_{(i,j+1)} \Big ){\textrm{d}} {\tilde{\mathbf{x}}}_{(i,j+1)}. \end{aligned}$$

We used EnKF as the computational method for the Bayesian filter. This marginalization can be performed by removing $\tilde{\textbf{x}}$ from the ensemble of the distribution $p({\textbf{x}}_{(i,j+1)}\mid \textbf{y}_{0:i-1},$ $\textbf{u}^*_{(0,1):(i,j)},\textbf{z}_{(i,j+1)})$. The control input $\textbf{u}_{(i,j+1)}^*$ is obtained as the expected value of the z-filtered distribution. The u-filter is applied by assimilating the estimated control input $\textbf{u}_{(i,j+1)}^*$ into the predicted distribution using the Bayesian filter based on Eq. (3). The control estimation process can proceed by repeating the following three steps: prediction$\rightarrow$z-filter$\rightarrow$u-filter.

The y-filter corresponds to the adaptive process and suppresses the uncertainties in the system model by assimilating the observations into the state distribution. Because the observation times $t_{i}$ differ from the latest u-filtered distribution $t_{i+1,0}$, the y-filter is executed by assimilating $\textbf{y}_i$ into the joint distribution of the state vectors at two time points. The ensemble approximating the joint distribution $p(\textbf{x}_{(i+1,0)},\textbf{x}_{(i,0)}\mid \textbf{y}_{0:i-1},\textbf{u}^*_{(0,1):(i+1,0)})$ can be obtained by concatenating the u-filtered ensemble at $t_{i+1,0}$ with the stored ensemble at $t_{i}$. The filtered distribution $p(\textbf{x}_{(i+1,0)},\textbf{x}_{(i,0)}\mid \textbf{y}_{0:i},\textbf{u}^*_{(0,1):(i+1,0)})$ can be calculated by assimilating $\textbf{y}_{i}$ with the concatenated ensemble using Eq. (4). The ensemble of $p(\textbf{x}_{(i+1,0)}\mid \textbf{y}_{0:i},\textbf{u}^*_{(0,1):(i+1,0)})$ is obtained by marginalizing $\textbf{x}_{(i,0)}$.

**Figure 2:**
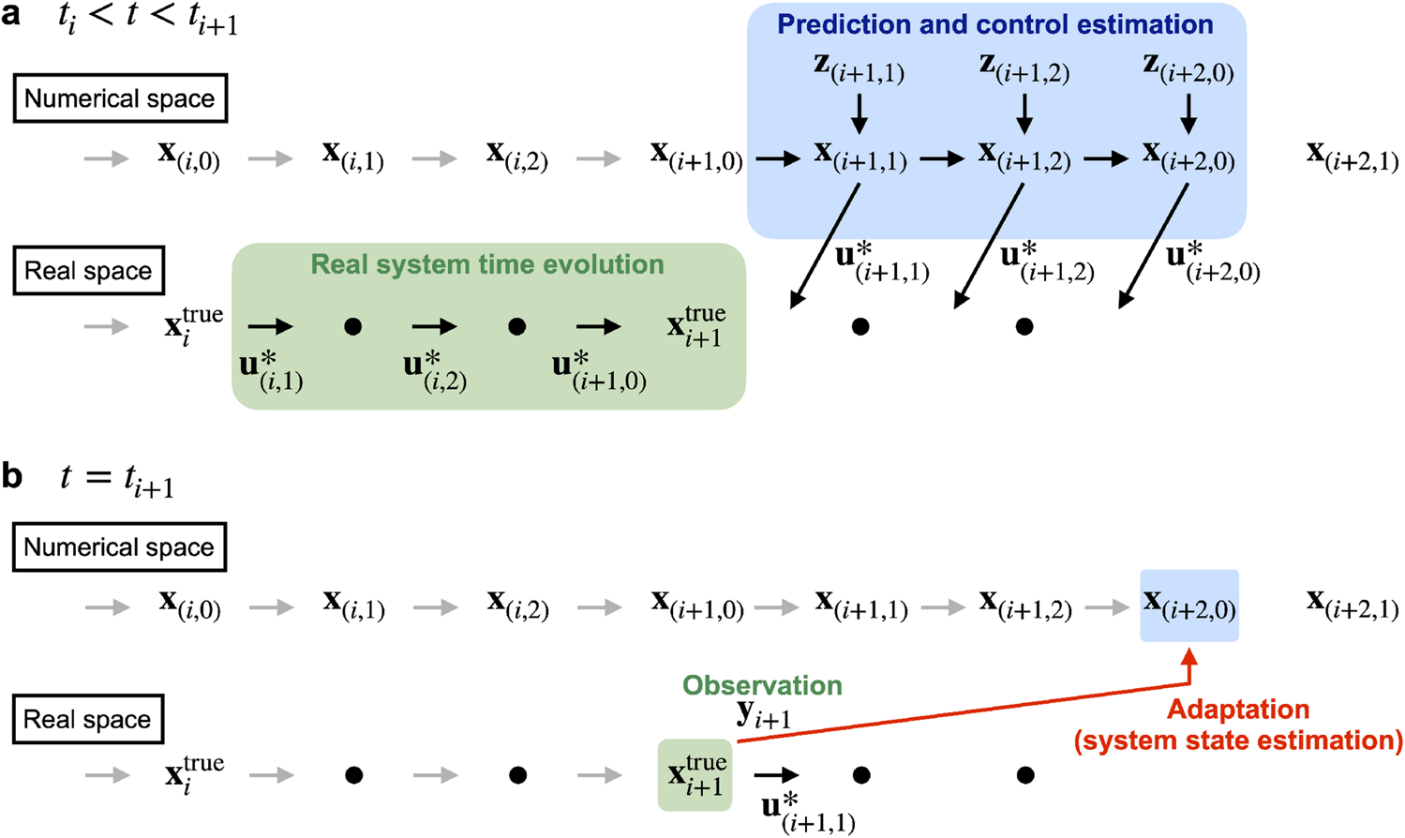
Control procedure of the LHD experiment ($\Delta t_y=3\Delta t_z$). The control is performed by repeating the control estimation ($\textbf{a}$) and DA of the observation into the latest u-filtered distribution ($\textbf{b}$).

This DA framework can be used to construct an adaptive predictive control algorithm without overlapping the prediction intervals. The control procedure for this experiment is summarized in Fig. 2. In $t_i< t < t_{i+1}$, while the real system evolves in time with the inputs $\textbf{u}_{(i,1)}^*$, $\textbf{u}_{(i,2)}^*$, and $\textbf{u}_{(i+1,0)}^*$, the predictions and control estimates are performed from $t_{i+1}$ to $t_{i+2}$ in the DA system, as shown in Fig. 2a. At $t = t_{i+1}$, the system state is observed as $\textbf{y}_{i+1}$, which is assimilated into the latest u-filtered distribution (adaptation), as shown in Fig. 2b. The loops of these processes illustrated in Fig. 2 provide an adaptive predictive control.

The proposed control system for LHD comprises a DA system “ASTI” (Assimilation System for Toroidal plasma Integrated simulation)^21^, which uses an integrated transport simulation code TASK3D^22,23^ as the system model for helical fusion plasmas and implements the EnKF and PF as DA techniques. TASK3D computes the heat and particle transport in a toroidal fusion plasma as a one-dimensional (1D) problem for the normalized minor radius. In this study, ASTI computed 256 ensemble members (TASK3D simulations) on a vector computer (NEC SX-Aurora TSUBASA, 16VE) connected to the LHD experimental system, and EnKF was used to perform the Bayesian filters. The computer had a maximum of 128 parallel processes, each of which was responsible for two ensemble members, thereby computing the 256 ensemble members. The control performance reached its maximum with 200 ensemble members in numerical experiments^18^ to control the virtual LHD plasma generated by TASK3D using noise values similar to those in the LHD experiments. Although the behavior of the virtual plasma is different from that of the actual plasma, the system model and state vector are almost the same as in the experiment. Therefore, the effect of the number of ensemble members on the control performance is considered to be sufficiently small in the LHD experiment.

### System model for LHD plasmas

We employed the TASK3D code^22,24^ as the time evolution model *f* in Eq. (1). TASK3D is an integrated simulation code for helical fusion plasmas that solves the 1D diffusive transport problem in the radial $\rho$-direction. The parameter $\rho$ is given by the magnetic flux surface, where 0 and 1 correspond to the center and edge of the plasma, respectively. We solved the heat transport equations for each electron and ion species in the LHD experiment. Assume that the electron and ion density profiles are similar, i.e., $n=n_{\textrm{e}}=n_{\textrm{i}}$. The radial profile in TASK3D was defined at 60 grid points. The geometric parameters required in the 1D transport simulation were evaluated by an equilibrium magnetic field precomputed using the VMEC code^25^ for the typical LHD magnetic configuration. The major radius of the magnetic axis in vacuum was 3.6 m and the magnetic-field strength at the plasma center was 2.75 T. In TASK3D, changes in the geometric parameters were not considered in the time evolution calculation due to the computational cost.

For the electron and ion thermal diffusivities, the following gyro-Bohm models were employed: $\chi _{\textrm{e}} = C^{\textrm{gB}}_{\textrm{e}}(T_{\textrm{e}}/{eB}) (\rho _{\textrm{i}}/{a})$ and $\chi _{\textrm{i}} = C^{\textrm{gB}}_{\textrm{i}}(T_{\textrm{i}}/{eB}) (\rho _{\textrm{i}}/{a})$. where *B*, $\rho _{\textrm{i}}$, and *a* are the magnetic field strength, ion Larmor radius, and plasma minor radius, respectively. We set $C^{\textrm{gB}}_{\textrm{e}}=1.5$ and $C^{\textrm{gB}}_{\textrm{i}}=0.1$ as reasonable values based on previous studies^24,26^. The heating power source comprised the externally applied ECH, power exchange between species, and loss term by interaction with neutrals. ECH only contributes to the heating term of the electron and the following ECH model was employed in the experiments:

13 $$\begin{aligned} p_{\textrm{ECH}}(\rho )= A \exp \left( -\frac{1}{2}\frac{(\mu _{\textrm{ECH}} - \rho )^2}{\sigma ^2_{\textrm{ECH}}} \right) , \end{aligned}$$

where $\mu _{\textrm{ECH}}=0.15$ and $\sigma _{\textrm{ECH}}=0.04$ are obtained from the ray-tracing calculations^27^. The coefficient *A* is determined from the total ECH input power, which is given by

14 $$\begin{aligned} P_{\textrm{ECH}}= \int _0^1 p_{\textrm{ECH}}(\rho ) \mathscr{V}'(\rho ) \textrm{d}\rho , \end{aligned}$$

where $\mathscr{V}$ is the plasma volume and $\mathscr{V}'=\textrm{d}\mathscr{V}/\textrm{d}\rho$.

### ECH control experiment

To demonstrate real-time adaptation and control estimation using the developed system, we considered a simple LHD experiment to control the central electron temperature by adjusting the ECH power^28,29^. Table 1 lists the state ($\tilde{\textbf{x}}$ and $\textbf{u}$), target state ($\textbf{z}$), and observation variables ($\textbf{y}$) used in the experiment. The radial profiles of the state variables were defined on 11 grid points ($\rho = 0, 0.1, 0.2, \ldots , 1$) in the state vector, and B-spline interpolation^30^ was used to transform the radial profiles to those on the TASK3D grid (60 grid points)^21^. The factors for the thermal diffusivities $c_{\textrm{e}}$ and $c_{\textrm{i}}$ were introduced to optimize $\chi _{\textrm{e}}$ and $\chi _{\textrm{i}}$ with large uncertainties. In the prediction step, $c_{\textrm{e}} \chi _{\textrm{e}}$ and $c_{\textrm{i}}\chi _{\textrm{i}}$ were used instead of $\chi _{\textrm{e}}$ and $\chi _{\textrm{i}}$ in the TASK3D simulation. The ECH used the four gyrotrons: 330, 400, 600, and 600 kW for 5.5 s; thus, ASTI controlled the injection power by selecting a subset of these gyrotrons by switching the individual gyrotrons on or off. ASTI sends the estimated control signals to the gyrotrons every $\Delta t_z =0.1$ s and receives the observed radial profiles of the electron temperature and density every $\Delta t_y =0.3$ s from the real-time Thomson scattering measurement system.

**Table 1:** State, target, and observation variables with their dimensions in the vectors ($M_i$).

Variable			$M_i$
$\tilde{\textbf{x}}$	*n*	Density	11
	$T_{\textrm{e}}$	Electron temperature	11
	$T_{\textrm{i}}$	Ion temperature	11
	$c_{\textrm{e}}$	Factor for electron thermal diffusivity	11
	$c_{\textrm{i}}$	Factor for ion thermal diffusivity	11
$\textbf{u}$	$P_{\textrm{ECH}}$	ECH input power	1
$\textbf{z}$	$T_{\textrm{e},\rho =0}$	Electron temperature at plasma center	1
$\textbf{y}$	*n*	Density	11
	$T_{\textrm{e}}$	Electron temperature	11

To ensure that the behavior of the system model resembles that of the real system at the beginning of control, observation assimilation was performed with a fixed heating power of 731 kW during the first phase $t<2.1$ s. ASTI assimilates the observations up to 1.8 s, after which the control estimation begins. Subsequently, the ECH power control commences at 2.1 s to produce the target electron temperature (4 keV) at 3.9 s and maintain it. The electron density at the plasma center was maintained at $1.5\times 10^{19}\ \textrm{m}^{-3}$ using the PID control to focus on the temperature control.

The covariance matrices for the noises $Q_{(i,j+1)}$, $R^\textbf{z}_{(i,j)}$, $R^\textbf{u}_{(i,j)}$, and $R^\textbf{y}_i$ are the key parameters affecting the overall control performance. In addition to them, the ensemble mean $\hat{\textbf{x}}_{(0,0)}$ and covariance matrix $V_{(0,0)}$ are required to generate the initial ensemble members. Diagonal matrices were used for these covariance matrices, i.e., the covariance component of the matrices was not considered.

The covariance matrix for the system noise, $Q_{(i,j+1)}$, controls the uncertainty of the system state, including the model parameters and control inputs. System noise was added before each prediction step, and $\tilde{\textbf{x}}$ was assigned a slightly larger value of noise after processing by the y-filter to prevent the distribution from shrinking. The standard deviation of the system noise was fixed at the values listed in Table 2. The values of the standard deviation for $T_{\textrm{e}}$, $T_{\textrm{i}}$, $c_{\textrm{e}}$, and $c_{\textrm{i}}$ were determined based on a previous study on data assimilation for the LHD plasmas^18,21^. The noises for $n_{\textrm{e}}$ were set to large values because ASTI did not compute the density transport. In control estimation, system noise for $\textbf{u}$ is an important parameter determining the range of control inputs considered in a single control estimation and the change rate of $\textbf{u}$. In this experiment, the standard deviation of the noise for $P_{\textrm{ECH}}$ was set to a sufficiently large value to track the rate of change of the target temperature.

**Table 2:** Standard deviations of the initial state distribution ($\sigma _{\textrm{init}}$) and system noise ($\sigma _{Q}$).

Variable		$\sigma _{\textrm{init}}$	$\sigma _{Q}$
$\tilde{\textbf{x}}$	*n*	20%	2% (10%)
	$T_{\textrm{e}}$	15%	2% (3%)
	$T_{\textrm{i}}$	15%	2% (3%)
	$c_{\textrm{e}}$	0.2	0 (0.1)
	$c_{\textrm{i}}$	0.2	0 (0.1)
$\textbf{u}$	$P_{\textrm{ECH}}$	0	0.5 (0)

The values in parentheses represent the additional system noise added after the y-filter. The values with% as the unit represent the rate for determining the standard deviation in proportion to the state distribution mean.

The covariance matrix $R^\textbf{z}_{(i,j)}$ affects the performance of the z-filter and determines the proximity of the system state to the target state after the z-filter step. The diagonal components of $R^\textbf{z}_{(i,j)}$ were determined at each z-filtering step as follows:

15 $$\begin{aligned} \left( R^\textbf{z}_{(i,j)} \right) _{ll} = r_{z}^2 \left( H^\textbf{z}{V_{(i,j)}}(H^\textbf{z})^{\textrm{T}} \right) _{ll}, \end{aligned}$$

where $V_{(i,j)}$ denotes the covariance matrix of the ensemble that approximates the predicted distribution at $t_{i,j}$ and $r_z=0.5$^18^. The subscript $(\ )_{ll}$ and superscript $^{\textrm{T}}$ denote the *l*-th diagonal component and matrix transposition, respectively.

The covariance matrix $R^{\textbf{u}}_{(i,j)}$ considers the uncertainty in the control input. In the experiment, the standard deviations of the control input noise were set to a sufficiently small value of 0.05 MW for $P_{\textrm{ECH}}$.

The covariance matrix $R^\textbf{y}_{i}$ affects the performance of the y-filter and determines the effect of the observed information on the state distribution. The standard deviation of the observation noise is assumed to be proportional to the difference between the observation and mean of the state distribution^18^,

16 $$\begin{aligned} \left( R^\textbf{y}_{i} \right) _{ll} = r_{y}^2 \left( \textbf{y}_i- H^\textbf{y}\hat{\textbf{x}}_{(i,0)} \right) _{l}^2, \end{aligned}$$

where $r_y=0.8$, $\hat{\textbf{x}}_{(i,0)}$ is the mean of the ensemble approximating $p(\textbf{x}_{(i,0)}\mid \textbf{y}_{0:i-1},\textbf{u}^*_{(0,1):(i,0)})$, and $(\ )_l$ represents the *l*-th element of the vector. This assumption prevents the control instability caused by overfitting of the system model to noisy observations and maintains the variance of the y-filtered ensemble at an adequate magnitude.

The initial ensemble means of $T_{\textrm{e}}$ and $T_{\textrm{i}}$ are set to the steady-state radial profiles calculated by the TASK3D simulation for the initial ECH. The initial mean profile of *n* is set to $n(\rho )=1.0-0.8\rho ^8$ [$\times 10^{19}\textrm{m}^{-3}$] and those of $c_{\textrm{e}}$ and $c_{\textrm{i}}$ are set to 1. The initial ensemble variances are assigned the values listed in Table 2.

### Real-time observation and control system

The DA system ASTI was connected to the real-time measurement system and ECH system^28,29^ via the real-time communication system in LHD^31^. The temperature and density profiles along with their measurement errors obtained from the Thomson scattering measurement system^32,33^ were sent from the Thomson data analysis PC to the vector engine server (SX-Aurora TSUBASA, NEC Inc.) via socket communication. The digitizers of the high-repetition-rate Thomson scattering system^34^ were used for real-time measurements (10 Hz). In the vector engine server, ASTI considers the time delay relative to real time, which is tens of milliseconds. This is attributed to the temperature computation, density analysis, and communication delay. The electron density and temperature were observed at 144 radial points, of which 66 data points were available for real-time measurement. After removing the obvious outliers based on measurement errors, a mapping model was used to transform the observed data in the real space coordinate (major radius *R*) into a form suitable for DA (profiles on 11 points of the $\rho$-coordinate).

The mapping model acted as coordinate transformation, outlier removal, data smoothing, and data point extraction for DA, providing the observation vector $\textbf{y}$ to ASTI. The mapping model was based on a multilayer perceptron with five hidden layers containing 500 units each using ReLu as the activation function. The mapping model was trained using data on the LHD experimental database (profiles observed by the Thomson scattering system and the mapped data obtained from the equilibrium calculations). The training data comprised 58 discharges (7769 time points) of the ECH plasma, of which a quarter were used as the test data. For all discharges in the training data, equilibrium magnetic fields were calculated and used to evaluate the density and temperature profiles in the $\rho$-coordinate for the teacher data. Before training, the mapped data were fitted with even-order polynomials up to the 8th order to smooth the profiles and extract the data points required for DA. An even function was used for fitting because the Neumann boundary condition $\partial /\partial \rho = 0$ was imposed on $\rho =0$ in TASK3D. The variations of the magnetic surfaces were considered in this mapping model, and the observations in the $\rho$-coordinate were provided to ASTI. Since TASK3D did not solve for the magnetic equilibrium, errors occurred in the geometric parameters required for the 1D transport simulation (e.g., $\textrm{d}\rho /\textrm{d}R$). In this experiment, these errors were considered small for helical devices and allowed. However, the real-time computation of the magnetic field, which is essential for tokamak control, will be addressed in future studies.

After the prediction and control estimation in ASTI were completed, the generated ECH control signals were sent to the computer (Jetson AGX Orin, NVIDIA Inc.) via socket communication. The digital signal for ECH control, which was converted to an analog signal using a D/A converter (EVAL-AD5686RSDZ, Analog Device Inc.) connected to a computer, was input to the ECH system to control the ON/OFF state of the ECH injection.

## Results

**Figure 3:**
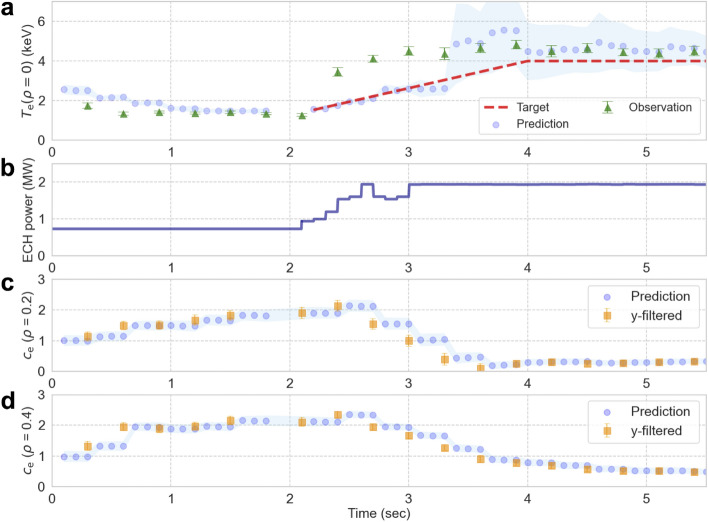
Results of a control experiment (#186500). ($\textbf{a}$) Control result of $T_{\textrm{e}}$ at the plasma center. The plotted values labeled “Prediction” correspond to the expected values of the predicted distribution for $t \le 2.1$ s and those of the u-filtered distributions for $t>2.1$ s. The shaded areas represent a single standard deviation of the distributions. The plotted values labeled “Observation” are those obtained by the mapping model from the Thomson scattering measurements. ($\textbf{b}$) ECH power adjusted using ASTI. ($\textbf{c}$) and ($\textbf{d}$) Distribution (expected value and one standard deviation) of ($c_{\textrm{e}}$) at $\rho =0.2$ and 0.4 used in the prediction step (“Prediction”) and y-filtered distribution (“y-filtered”).

**Figure 4:**
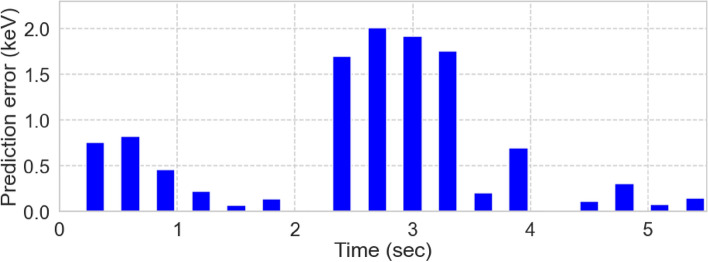
Prediction error defined as the absolute difference between “Prediction” and “Observation” in Fig. 3.

Figure 3 shows the control results of the LHD experiment and Fig. 4 shows the prediction error (the absolute difference between “Prediction” and “Observation” in Fig. 3). The electron temperature increases to that of the target (4 keV) and is maintained beyond  3.9 s. The prediction error, shown in Fig. 4, increases to 2 keV in the transient section (2.1 s $\le t<$ 3.9 s) and decreases to approximately 0.2 keV in the steady-state section (3.9 s $\le t$). These results demonstrate the effectiveness of real-time adaptation and control estimation using the DA-based control system. The prediction error in the transient section occurred mainly because the gyro-Bohm model overestimated the thermal diffusivities as the electron temperature increased. At the beginning of the control (23 s), the $c_{\textrm{e}}$ optimization lagged behind the variation in the actual thermal diffusivities. However, before 4 s, the gap between the model and the actual system was bridged, which improved the prediction performance. The prediction error also depends on the Thomson measurement error of 0.2–0.4 keV and accuracy of the mapping model.

In addition, the discrete nature of the control input and expansion of the u-filtered distribution from 3.4 s onward also affect the control accuracy. This expansion is attributed to the increase in ensemble members with small $c_{\textrm{e}} (<0.3)$ near the center as shown in Fig. 3c, which increases the range of electron temperature variation. Because the state distribution is approximated by finite ensemble members, a large uncertainty in the predicted distribution makes it difficult to identify the relationship between the control inputs and the system state and may reduce the control accuracy. The expansion of the u-filtered distribution at 3.3 s was triggered by adding system noise with the fixed standard deviation of 0.1 to $c_{\textrm{e}}$, despite the small mean of $c_{\textrm{e}}$ near the center. This expansion can be avoided by introducing a method to dynamically adjust the system noise by considering the characteristics of the system model. The probability distribution of model parameters should be adjusted by considering the physical characteristics and control accuracy. This issue will be addressed in a subsequent control experiment.

### Change of state distribution in the control process

**Figure 5:**
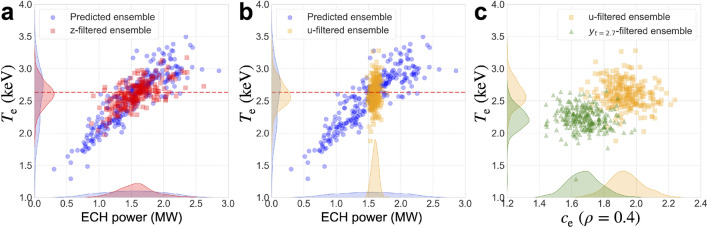
Variations in the ensembles that approximate the state distributions at $t=3.0$ s in the control experiment. (**a**) Estimation of the control input (z-filter). (**b**) Predicted distribution modified by the estimated control input (u-filter). (**c**) Adaptation to the actual LHD plasma by assimilating the observation at $t=2.7$ s (y-filter).

The ensemble members of the state distributions at 3.0 s are shown in Fig. 5. Figure 5a shows the z-filter process in which the target temperature is assimilated into the predicted ensemble. The z-filtered ensemble approximates the distribution $p(\textbf{x}_{3.0} \mid \textbf{y}_{0.3:2.4}, \textbf{u}^*_{2.1:2.9}, \textbf{z}_{3.0})$, where the subscript number indicates the time (s) and $t_1:t_2$ denotes the values in $[t_1,t_2]$. As shown in Fig. 5b, the u-filtered ensemble is computed after the control input $\textbf{u}_{3.0}^*$ is obtained as the expected value of this distribution. The distribution approximated by the u-filtered ensemble corresponds to the predicted distribution with zero or small uncertainty in the control input. The remaining uncertainties originate from the uncertainties in the model parameters and system state before time evolution calculation. The u-filtered ensemble is modified by assimilating the observation $\textbf{y}_{2.7}$ (y-filter), as shown in Fig. 5c. This filter optimizes the state variables, including the model parameters, to adapt the system model to the real system. The control process proceeds to the subsequent prediction from the y-filtered ensemble.

The filtering steps were performed using EnKF in the simple control experiment. Strongly nonlinear relationships among the state variables and a significantly non-Gaussian state distribution (e.g., a multimodal distribution) necessitate the use of other filters (e.g., PF) and another definition of $\textbf{u}^*$ (e.g., mode).

### Real-time adaptation to the real system

**Figure 6:**
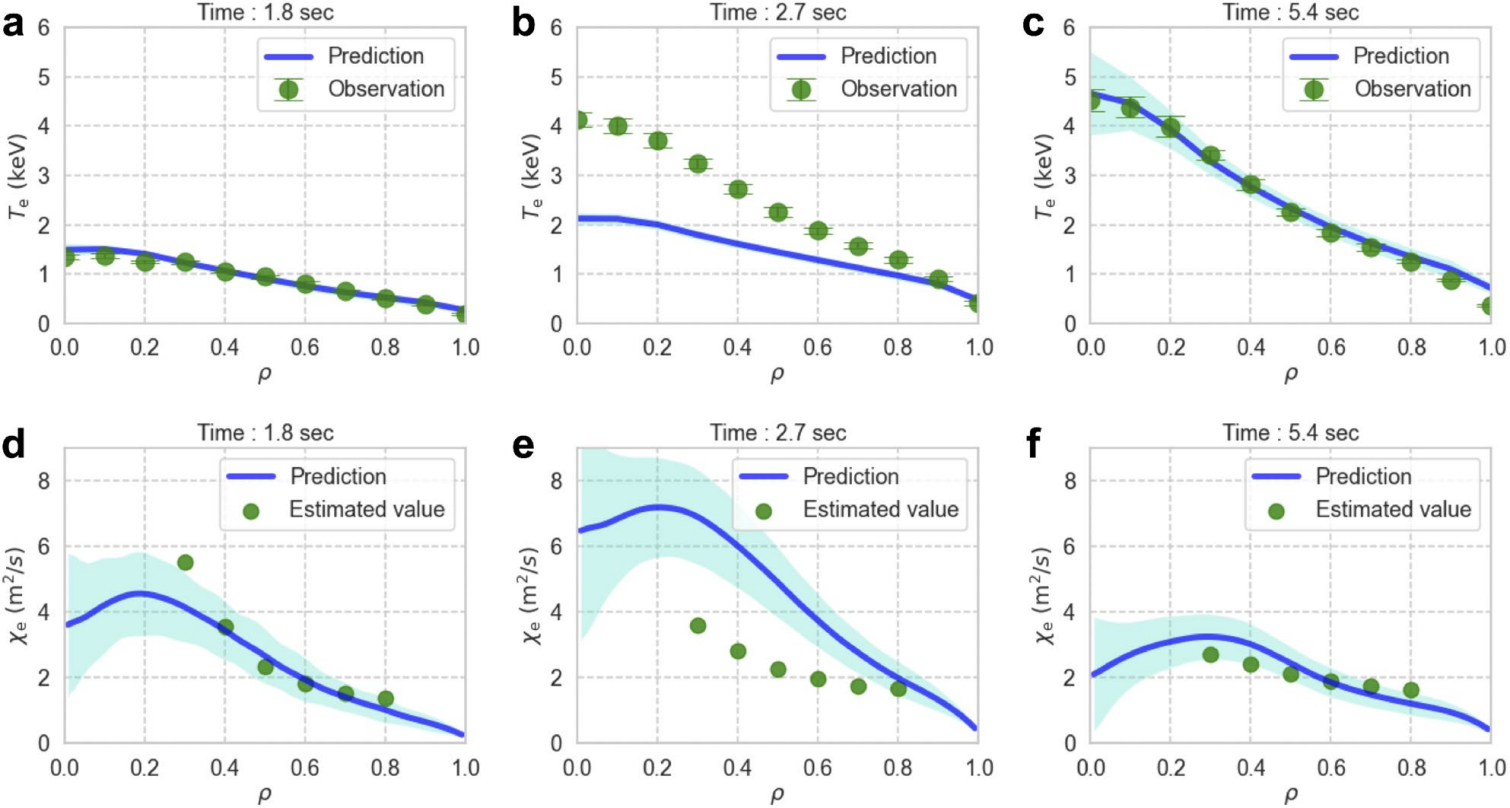
Time evolution of the radial profiles of electron temperature and thermal diffusivity. (**a**–**c**) Radial profiles of the predicted electron temperature corresponding to Fig. 3a and the observations (mapped to the $\rho$-coordinate) at three points in time. (**d**–**f**) Radial profiles of electron thermal diffusivity calculated in the prediction step (“Prediction”) and the values estimated using Eq. (17) from the observed temperature profile at the corresponding time points (“Estimated value”). The shaded areas around the radial profiles represent a single standard deviation.

The upper panels of Fig. 6 show the radial profiles of the predicted electron temperature (u-filtered distribution) and observed profiles at three different points in time. The radial profiles of the electron thermal diffusivity are shown in the lower panels. The thermal diffusivity can be estimated from the observed density and temperature profiles as

17 $$\begin{aligned} \chi (\rho ) \sim \frac{-\frac{1}{\mathscr {V}'}\int ^{\rho }_{0} p_{\textrm{ECH}}\mathscr{V}'\textrm{d}\rho }{\langle |\nabla \rho |^2 \rangle n \frac{\partial T}{\partial \rho }}, \end{aligned}$$

where $\langle \ \rangle$ represents the magnetic flux surface average. This estimate is less valid near the center where the temperature gradient is smaller.

After the first phase (observation assimilation), the temperature profile was predicted with high accuracy at the beginning of control estimation (t = 1.8 s), as shown in Fig. 6a,d. In the transient phase (2.1 s $\le t<$ 3.9 s), insufficient modeling of the electron thermal diffusivity (Fig. 6e) underestimated the profile around the plasma center, as shown in Fig. 6b. These prediction errors contribute to the control error in the transient phase, as shown in Fig. 3. However, in the steady-state phase (3.9 s $\le t$), the temperature profile was predicted with high accuracy by optimizing the thermal diffusivity model, as shown in Fig. 6c,f.

These results demonstrate the real-time adaptation of the integrated transport simulation code to the actual LHD plasma. In the transient phase, changes in the circumstances and the discrepancy between the system model and real system degrade the prediction performance. Furthermore, in the control procedure used in this experiment, the observations obtained at time $t_i$ were reflected in the control estimation after $t_i+\Delta t_y$, which caused a delay in adaptation, especially at the beginning of the control (2.1 s−). This delay can be a critical issue for some control problems. Therefore, to achieve a stable adaptive predictive control system, it is important to design the control procedure, $\Delta t_y$, and the observation noise to minimize this delay.

Reduction of the control error in the transient phase makes it necessary to improve the assumed transport model, adjust the adaptive capacity by controlling the uncertainty in model parameters, and minimize the adaptation delay. These limitations will be addressed in the future studies aimed at building a stable control system. The DA framework can be applied to construct an advanced control system that can optimize the timing, physical quantities, and measurement positions. For instance, it would be possible to develop a system that allows measurement devices to observe the system state depending on the uncertainty of the model parameters and system state.

## Discussion

A control system based on DA was developed to control LHD plasmas. A simple experiment to control the electron temperature demonstrated adaptive predictive control using a nonlinear system model (integrated transport model) with large uncertainties. The proposed control framework simplifies the structure of the control system to accommodate complex control problems characterized by a large number of variables.

The adaptive capacity of a control system is limited by the assumed models and frequency of observations^18^. Therefore, the sophistication of the model, computational cost, and amount of information contained in the observations must be adjusted depending on the target control problem. Similar to other control systems, observation errors significantly affect the performance of the proposed DA-based control system. However, this system can explicitly account for the uncertainties in observations based on the observation noise. Robust and stable control can be achieved by exploiting this uncertainty; however, methods to adjust the uncertainties in observations and model parameters according to the situation should be developed.

Advanced control systems constructed for future fusion reactors would require models based on data-driven approaches, such as surrogate models^35–37^, stability indicators^15,38,39^, and tomography methods^40,41^. To this end, the DA-based control system, which expresses the system state as a probability distribution, is highly compatible with other data-driven approaches and provides a flexible control platform that links the physics-based and data-driven models.

The results of the first application of DA-based control system in LHD do not exceed the control performance of conventional controllers (e.g., PID controller). However, the proposed approach has high potential in the control of future fusion reactors under large uncertainties because it can estimate and control the internal state from limited observed information and naturally handle various observed and control variables (e.g., temperature and density profiles) and plasma responses with different time scales in a single framework. Thus, the success of this demonstration paves the way for addressing challenging control problems in fusion plasma, such as multivariate control, radial profile control, electric field bifurcation control, and avoidance of terminating events using relevant alarm rates. DA-based control may become a key technology for solving challenging control problems in future fusion reactors and other complex systems by augmenting numerical models with real systems. Beginning in 2024, our roadmap includes conducting more sophisticated control experiments with this control system by incorporating additional measurement, heating, and fuel-supply devices into the system. Additionally, we are actively working on extending our control capabilities to include Tokamak control.

## Data Availability

The raw data were generated at the LHD facility. Automatic Integrated Data Analysis software and the analyzed data are available from the LHD data repository (https://doi.org/10.57451/lhd.analyzed-data). The calculated data depicted in the figures of this paper are available from the corresponding author upon reasonable request.

## References

[1] Fasoli A, Brunner S, Cooper W, Graves J, Ricci P, Sauter O, Villard L (2016). Computational challenges in magnetic-confinement fusion physics. Nat. Phys..

[2] Winter J (1996). Wall conditioning in fusion devices and its influence on plasma performance. Plasma Phys. Control. Fusion.

[3] Mayne DQ (2014). Model predictive control: Recent developments and future promise. Automatica.

[4] Gettelman A, Geer AJ, Forbes RM, Carmichael GR, Feingold G, Posselt DJ, Stephens GL, van den Heever SC, Varble AC, Zuidema P (2022). The future of earth system prediction: Advances in model-data fusion. Sci. Adv..

[5] Carrassi A, Bocquet M, Bertino L, Evensen G (2018). Data assimilation in the geosciences: An overview of methods, issues, and perspectives. Wiley Interdiscip. Rev. Climate Change.

[6] King J, Anchukaitis KJ, Allen K, Vance T, Hessl A (2023). Trends and variability in the southern annular mode over the common era. Nat. Commun..

[7] Tierney JE, Zhu J, Li M, Ridgwell A, Hakim GJ, Poulsen CJ, Whiteford RD, Rae JW, Kump LR (2022). Spatial patterns of climate change across the paleocene-eocene thermal maximum. Proc. Natl. Acad. Sci..

[8] Hou X, Gao S, Li Q, Kang Y, Chen N, Chen K, Rao J, Ellenberg JS, Patz JA (2021). Intracounty modeling of Covid-19 infection with human mobility: Assessing spatial heterogeneity with business traffic, age, and race. Proc. Natl. Acad. Sci..

[9] Kato H, Yoshizawa A, Ueno G, Obayashi S (2015). A data assimilation methodology for reconstructing turbulent flows around aircraft. J. Comput. Phys..

[10] Tian S, Van Dijk AI, Tregoning P, Renzullo LJ (2019). Forecasting dryland vegetation condition months in advance through satellite data assimilation. Nat. Commun..

[11] Miyoshi T, Kunii M, Ruiz J, Lien G-Y, Satoh S, Ushio T, Bessho K, Seko H, Tomita H, Ishikawa Y (2016). “Big data assimilation” revolutionizing severe weather prediction. Bull. Am. Meteor. Soc..

[12] Tian S, Van Dijk AI, Tregoning P, Renzullo LJ (2019). Forecasting dryland vegetation condition months in advance through satellite data assimilation. Nat. Commun..

[13] Degrave J, Felici F, Buchli J, Neunert M, Tracey B, Carpanese F, Ewalds T, Hafner R, Abdolmaleki A, de Las Casas D (2022). Magnetic control of tokamak plasmas through deep reinforcement learning. Nature.

[14] Wakatsuki T, Suzuki T, Oyama N, Hayashi N (2021). Ion temperature gradient control using reinforcement learning technique. Nucl. Fusion.

[15] Kates-Harbeck J, Svyatkovskiy A, Tang W (2019). Predicting disruptive instabilities in controlled fusion plasmas through deep learning. Nature.

[16] Takeiri Y, Morisaki T, Osakabe M, Yokoyama M, Sakakibara S, Takahashi H, Nakamura Y, Oishi T, Motojima G, Murakami S (2017). Extension of the operational regime of the LHD towards a deuterium experiment. Nucl. Fusion.

[17] Osakabe M, Takahashi H, Yamada H, Tanaka K, Kobayashi T, Ida K, Ohdachi S, Varela J, Ogawa K, Kobayashi M (2022). Recent results from deuterium experiments on the large helical device and their contribution to fusion reactor development. Nucl. Fusion.

[18] Morishita Y, Murakami S, Yokoyama M, Ueno G (2023). Data assimilation and control system for adaptive model predictive control. J. Comput. Sci..

[19] Evensen G (2003). The ensemble kalman filter: Theoretical formulation and practical implementation. Ocean Dyn..

[20] Kitagawa G (1996). Monte Carlo filter and smoother for non-gaussian nonlinear state space models. J. Comput. Graph. Stat..

[21] Morishita Y, Murakami S, Yokoyama M, Ueno G (2022). ASTI: Data assimilation system for particle and heat transport in toroidal plasmas. Comput. Phys. Commun..

[22] Murakami S, Yamaguchi H, Sakai A, Wakasa A, Fukuyama A, Nagaoka K, Takahashi H, Nakano H, Osakabe M, Ida K (2015). Integrated transport simulations of high ion temperature plasmas of LHD. Plasma Phys. Control. Fusion.

[23] Yokoyama M, Seki R, Suzuki C, Sato M, Emoto M, Murakami S, Osakabe M, Tsujimura TI, Yoshimura Y, Ido T (2017). Extended capability of the integrated transport analysis suite, task3d-a, for LHD experiment. Nucl. Fusion.

[24] Sakai A, Murakami S, Yamaguchi H, Wakasa A, Fukuyama A, Nagaoka K, Takahashi H, Nakano H, Osakabe M (2015). Integrated particle transport simulation of NBI plasmas in LHD. Plasma Fusion Res..

[25] Hirshman S, Merkel P (1986). Three-dimensional free boundary calculations using a spectral green’s function method. Comput. Phys. Commun..

[26] Wakasa, A., Fukuyama, A., Murakami, S., Miki, M., Yokoyama, M., Sato, M., Toda, S., Funaba, H., Tanaka, K., Ida, K., Yamada, H., Honda, M., & Nakajima, N. Integrated transport simulation of LHD plasmas using TASK3D, in *Proc. 23rd IAEA Fusion Energy Conf. (Daejon)*, P4.029 (2010).

[27] Tsujimura TI, Kubo S, Takahashi H, Makino R, Seki R, Yoshimura Y, Igami H, Shimozuma T, Ida K, Suzuki C (2015). Development and application of a ray-tracing code integrating with 3d equilibrium mapping in LHD ECH experiments. Nucl. Fusion.

[28] Takahashi H, Shimozuma T, Kubo S, Yoshimura Y, Igami H, Ito S, Kobayashi S, Mizuno Y, Okada K, Mutoh T (2014). Extension of high t e regime with upgraded electron cyclotron resonance heating system in the large helical device. Phys. Plasmas.

[29] Shimozuma T, Takahashi H, Kubo S, Yoshimura Y, Igami H, Takita Y, Kobayashi S, Ito S, Mizuno Y, Idei H (2010). Ecrh-related technologies for high-power and steady-state operation in LHD. Fusion Sci. Technol..

[30] De Boor C (1978). A Practical Guide to Splines.

[31] Nakanishi H, Ohsuna M, Kojima M, Imazu S, Nonomura M, Emoto M, Yoshida M, Iwata C, Ida K (2016). Real-time data streaming and storing structure for the LHD’s fusion plasma experiments. IEEE Trans. Nucl. Sci..

[32] Narihara K, Yamada I, Hayashi H, Yamauchi K (2001). Design and performance of the Thomson scattering diagnostic on LHD. Rev. Sci. Instrum..

[33] Yamada, I., Narihara, K., Funaba, H., Minami, T., Hayashi, H., Kohmoto, T., Group, L.E. (2010). Recent progress of the LHD Thomson scattering system. Fusion Sci. Technol..

[34] Funaba H, Yasuhara R, Uehara H, Yamada I, Sakamoto R, Osakabe M, Den Hartog D (2022). Electron temperature and density measurement by Thomson scattering with a high repetition rate laser of 20 khz on lhd. Sci. Rep..

[35] Narita E, Honda M, Nakata M, Yoshida M, Hayashi N (2021). Quasilinear turbulent particle and heat transport modelling with a neural-network-based approach founded on gyrokinetic calculations and experimental data. Nucl. Fusion.

[36] Rodriguez-Fernandez P, Howard N, Candy J (2022). Nonlinear gyrokinetic predictions of SPARC burning plasma profiles enabled by surrogate modeling. Nucl. Fusion.

[37] Dong G, Wei X, Bao J, Brochard G, Lin Z, Tang W (2021). Deep learning based surrogate models for first-principles global simulations of fusion plasmas. Nucl. Fusion.

[38] Murari A, Rossi R, Lungaroni M, Baruzzo M, Gelfusa M (2021). Stacking of predictors for the automatic classification of disruption types to optimize the control logic. Nucl. Fusion.

[39] Yokoyama T, Yamada H, Masuzaki S, Miyazawa J, Mukai K, Peterson BJ, Tamura N, Sakamoto R, Motojima G, Ida K (2020). Prediction of radiative collapse in large helical device using feature extraction by exhaustive search. J. Fusion Energy.

[40] Kenmochi N, Nishiura M, Nakamura K, Yoshida Z (2019). Tomographic reconstruction of imaging diagnostics with a generative adversarial network. Plasma Fusion Res..

[41] Carvalho D, Ferreira D, Carvalho P, Imrisek M, Mlynar J, Fernandes H, Contributors J (2019). Deep neural networks for plasma tomography with applications to jet and compass. J. Instrum..

